# Paradoxical Aortic Stenosis: Simplifying the Diagnostic Process

**DOI:** 10.5935/abc.20180075

**Published:** 2018-05

**Authors:** Vitor Emer Egypto Rosa, João Ricardo Cordeiro Fernandes, Antonio Sergio de Santis Andrade Lopes, Roney Orismar Sampaio, Flávio Tarasoutchi

**Affiliations:** Instituto do Coração (InCor) - Faculdade de Medicina da Universidade de São Paulo, São Paulo, SP - Brazil

**Keywords:** Aortic Valve Stenosis, Echocardiography, Aortic Valve

Severe aortic stenosis (AS) is defined as a significant reduction of the aortic valve area (aortic valve area [AVA] ≤ 1.0 cm^2^) associated with evidence of left ventricular hypertrophic response (aortic jet velocity > 4 m/s or mean gradient between the left ventricle and the aorta > 40 mmHg).^1^^-^3 However, as Minners et al.^4^ have demonstrated, inconsistencies in echocardiographic measurements are extremely frequent in daily clinical practice. In about 30% of the cases evaluated by AS, we found AVA ≤ 1.0 cm^2^, indicative of severe AS, with a mean gradient < 40 mmHg, suggestive of moderate AS.^4^ This dissociation makes it difficult to establish an adequate and definitive diagnosis to the patient with AS, fundamental point in the therapeutic decision making. If, on the one hand, patients with moderate AS do not benefit from valve intervention, those with severe AS require surgical aortic valve replacement or a transcatheter aortic bioprosthesis implant, especially if they are symptomatic.^1^^-^^3^

In 2007, Hachicha et al.,^5^ in a pioneering work, defined such patients as having "paradoxical AS" (or low-flow, low-gradient AS with preserved ejection fraction). These patients present a pathophysiology similar to that of diastolic heart failure, with hypertrophy and left ventricular compliance reduction, leading to a "low-flow" state, defined by an ejected volume (stroke volume) of < 35 ml/m^2^ (stroke volume = Diastolic Volume - Systolic Volume / Body Surface).^5^^-^^7^

Another important contribution of Hachicha et al^5^, corroborated by some subsequent studies,^8^^-^^11^ was the demonstration of a better survival of symptomatic patients with paradoxical AS after valve intervention when compared to clinical treatment. However, patients with paradoxical AS, despite being benefited by valve intervention, present higher surgical mortality when compared with patients with classic AS (mean gradient > 40mmHg).^1^^-^^3^^,^^8^^,^^9^^,^^11^

**Figure 1:**
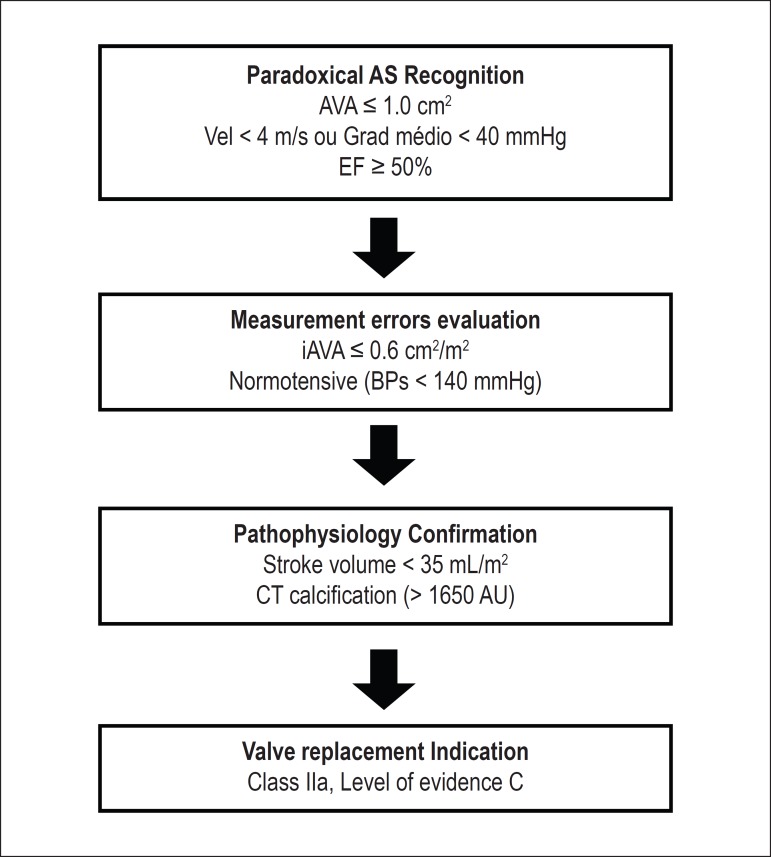
Algorithm proposed for the diagnosis of paradoxical aortic stenosis. * In patients with BMI above 30 kg/m^2^, we must use 0.5 cm^2^/m^2^ value as reference for iAVA. AS: aortic stenosis; AVA: aortic valve area; Vel: jet velocity; Grad: gradient; EF: ejection fraction; iAVA: indexed aortic valve area; sBP: systolic blood pressure; CT: computed tomography.

In this paper, we propose an algorithm to facilitate the diagnostic confirmation of paradoxical AS. In three steps, we perform the Recognition of Paradoxical AS, Measurement Error Evaluation and Pathophysiological Confirmation (Figure 1):

1. Recognition of Paradoxical AS: this step is the first and most important. The delay in the diagnosis of paradoxical AS causes delayed intervention, leading to an increase in mortality. The classification of "moderate to severe" or even "moderately-severe" valvulopathy is not described in any of the current guidelines and impairs clinical reasoning.^1^^-^^3^ For this reason, patients with AVA ≤ 1.0 cm^2^, jet velocity < 4 m/s or mean gradient < 40 mmHg and ejection fraction > 50% should be classified as having paradoxical AS or low-flow, low-gradient AS with preserved ejection fraction.
2. Evaluation of Measurement Errors: In this stage, we must identify eventual measurement errors that justify an underestimated gradient or AVA. The echocardiographer should be aware of the correct alignment of the Doppler continuous wave for velocity and gradient measurement, avoiding underestimating these measurements. Another orientation is to avoid AVA measurement by continuity equation and using whenever possible measurement by planimetry. AVA measurement by continuity equation may underestimate AVA, since such measurement takes into account left ventricular outflow tract area calculation (VSVE) (AVA = area of VSVE x VTI of VSVE/VTI of aortic valve; where VTI is time-velocity integral). The VSVE dimension is usually measured with a 2D echocardiogram, assuming that the VSVE is circular. However, such a structure can often be elliptical, causing measurement errors.^7^ 3D echocardiogram is a promising test for more accurate evaluation of VSVE and AVA by planimetry, however, specific studies for the population with paradoxical AS are necessary for its routine indication. Two points are extremely important for the clinical cardiologist. First, in patients with small corporeal surface, a reduced AVA may correspond to moderate AS. In this way we must always index AVA by the corporeal surface (iAVA), being that an iAVA ≤ 0.6 cm^2^/m^2^ suggests important AS. In obese patients (BMI ≥ 30 kg/m^2^) we must assume a lower cut-off value (< 0.5 cm^2^/m^2^) so as not to overestimate the anatomical severity.^12^ The second data that should be evaluated is systolic blood pressure in gradient measurement moment, which should be less than 140 mmHg.^1^ Higher pressures contribute to underestimating the mean gradient and generate an increase in the valvulo-arterial impedance, a measure that estimates the ventricular afterload added to arterial and valvular overload ventricle, and it is also associated with mortality.^13^ In summary, the clinical cardiologist should remember to index the AVA and make sure that the systolic blood pressure was < 140 mmHg at the time of gradient measurement, while the echocardiographer should be attentive to errors in gradient measurement and measure the AVA by the planimetry.
3. Pathophysiology Confirmation: Finally, we must confirm the pathophysiology of AS and low-flow, low-gradient. In developed countries, the main etiology of AS is degenerative, also known as calcific. Valvular calcification correlates with anatomic severity and values greater than 1650 AU, verified by computed tomography, suggest anatomically severe AS.^14^ However, females may present the same anatomic severity as men, but with lower values of calcification, being advised to apply differentiated cutoff values for female patients (> 1200 AU).^15^ Pathophysiology of low flow should be confirmed by stroke volume calculation, as previously described. In order to justify low gradient in a patient with severe AS, he must necessarily present a small cavity with stroke volume < 35 ml/m^2^.^1^^-^^3^^,^^5^^-^^7^

Thus, through this 3 steps algorithm, we help in the recognition of paradoxical AS anatomical severity, facilitating the clinician to identify the ideal moment for intervention in this difficult diagnosis entity.
